# Severe traumatic injury during long duration spaceflight: Light years beyond ATLS

**DOI:** 10.1186/1752-2897-3-4

**Published:** 2009-03-25

**Authors:** Andrew W Kirkpatrick, Chad G Ball, Mark Campbell, David R Williams, Scott E Parazynski, Kenneth L Mattox, Timothy J Broderick

**Affiliations:** 1 Foothills Medical Centre, 1403 29^th ^Street NW, Calgary, Alberta, T2N 2T9, USA; 2 Paris Regional Medical Center, 820 Clarksville St., Paris, Texas, 75460, USA; 3 NASA Johnson Space Center, 2101 NASA Pkwy #1, Houston, Texas, 77058, USA; 4 Baylor College of Medicine, Dept. of Surgery, One Baylor Pl., Houston, Texas, 77030, USA; 5 University of Cincinnati, Dept. of Surgery, 222 Piedmont Ave, #7000, Cincinnati, Ohio, 45219, USA

## Abstract

Traumatic injury strikes unexpectedly among the healthiest members of the human population, and has been an inevitable companion of exploration throughout history. In space flight beyond the Earth's orbit, NASA considers trauma to be the highest level of concern regarding the probable incidence versus impact on mission and health. Because of limited resources, medical care will have to focus on the conditions most likely to occur, as well as those with the most significant impact on the crew and mission. Although the relative risk of disabling injuries is significantly higher than traumatic deaths on earth, either issue would have catastrophic implications during space flight. As a result this review focuses on serious life-threatening injuries during space flight as determined by a NASA consensus conference attended by experts in all aspects of injury and space flight.

In addition to discussing the impact of various mission profiles on the risk of injury, this manuscript outlines all issues relevant to trauma during space flight. These include the epidemiology of trauma, the pathophysiology of injury during weightlessness, pre-hospital issues, novel technologies, the concept of a space surgeon, appropriate training for a space physician, resuscitation of injured astronauts, hemorrhage control (cavitary and external), surgery in space (open and minimally invasive), postoperative care, vascular access, interventional radiology and pharmacology.

Given the risks and isolation inherent in long duration space flight, a well trained surgeon and/or surgical capability will be required onboard any exploration vessel. More specifically, a broadly-trained surgically capable emergency/critical care specialist with innate capabilities to problem-solve and improvise would be desirable. It will be the ultimate remote setting, and hopefully one in which the most advanced of our societies' technologies can be pre-positioned to safeguard precious astronaut lives. Like so many previous space-related technologies, these developments will also greatly improve terrestrial care on earth.

## Introduction

Despite significant operational and economic challenges, the International Space Station (ISS) has been continuously manned for over eight years. Although it serves primarily as a human research facility in low earth orbit (LEO), on-board medical care is extremely limited. As a result, all patients with serious injuries will be evacuated to earth as soon as possible. The National Aeronautic and Space Administration (NASA) also envisions extended-duration exploration missions beyond LEO (Exploration Class Missions (ECM)) in the near future [1-3]. These plans were recently supported by the outgoing President of the United States, who stated an intention to pursue ECMs as early as 2015. A serious traumatic injury on such a mission would require in-flight treatment because a return to earth in less than 9 months would be unlikely for any manned flight to Mars [4,5]. Furthermore, in the absence of dramatic new technological developments, the space medicine community will continue to be limited by logistical considerations in weight, volume, power, and Crew Medical Officer (CMO) training [4,6].

Although the specifics of crew selection for future ECMs are still debated, astronauts will be screened extensively for chronic and inheritable diseases [3,7]. Unfortunately, traumatic injury strikes unexpectedly among the healthiest members of the human population, and has been an inevitable companion of exploration throughout history. In 1994, Billica and colleagues [8] ranked traumatic injury at the highest level of concern regarding the probable incidence versus impact on mission and health. This risk assessment reflects similar occurrences in analog space environments [9], as well as among enlisted men aboard US submarines where injury was both the leading cause of morbidity and time off of duty [10].

To date the most frequent medical incidents in the NASA-Mir orbital space program have involved minor traumatic injuries to the skin and mucus membranes [11]. Although numerous catastrophic deaths have occurred, there have been no evacuations for serious trauma in space. Because of limited resources, medical care will have to focus on the conditions most likely to occur, as well as those with the most significant impact on the crew and mission. Recognizing that there are at least 3 disabling injuries for every traumatic death on earth [12], any of which would have catastrophic implications for a ECM, this review will focus on serious life-threatening injuries. It is also implicit that any mission equipment available for trauma care would also have to be effective for other life-threatening general surgical, medical, and/or gynecologic emergencies.

### Space Mission Profiles and the Epidemiology of Trauma

Reviews of terrestrial trauma deaths have shown that a limited number of conditions cause the majority of preventable injury mortality [13-15]). These issues represent the focus of the initial resuscitative measures in the Advanced Trauma Life Support (ATLS) course of the American College of Surgeons. More specifically, ATLS aims to prevent unnecessary death from airway obstruction, hemo-pneumothoraces, circulatory instability (predominantly hemorrhage), and intra-cranial hemorrhage [12]. Although severe traumatic brain injury is the leading specific cause of trauma deaths in North America [16], effective treatments for primary brain injury remain limited [17]. As a result, airway obstruction, hemo-pneumothoraces, and bleeding are a critical focus for space trauma planning.

The intended mission profile and crew duties will greatly influence the relative risk and approach to treatment for injury in space. They will also dictate complicated algorithms outlining continuity of care. These will potentially involve emergency extra-terrestrial care at the point of injury, stabilization for space-evacuation, space transport (involving challenges of ascent, entry and landing), terrestrial land site resuscitation and treatment, terrestrial transportation, and eventually definitive care of all injuries. Although delivering definitive medical care in space would obviate many of the transportation related dangers, it would also greatly increase capability requirements. More specifically, long duration space experience may involve intervals in LEO aboard the ISS, traveling to the Moon or Mars, and/or residence in the reduced gravity environments of either destination. Currently on the ISS, prolonged times for deployment of stowed equipment would further complicate any emergent resuscitation. LEO, and possibly the Moon, would however offer the potential for telemedicine support, damage control interventions, and definitive therapy via either a pre-configured care facility, or damage control evacuation to earth. Although the original plans for serious injuries in LEO specified an early return to earth, this paradigm is complicated by the limited life-span of the Space Shuttle Program, as well as the cancellation of a dedicated emergency crew return vehicle [18]. As a result, ECMs will likely require complete autonomy and redundancy.

In addition to the mission goals, potential construction on a Martian or Lunar surface, as well as the area of surface exploration (extra-vehicular activity (EVA)) will be critical parameters that influence the nature of trauma and the potential for survival. Penetrating injuries from micrometeorites may occur when an astronaut is exposed outside the spacecraft. Such trauma might be associated with failure of the space-suit, resulting in either a flash fire or explosive decompression to the vacuum of space. In either situation the chances of survival would be nil [6,19]. More probable is the potential for crush-type injuries[6,20,21]. Although objects are weightless in space, they retain mass and can therefore generate significant forces [22]. In the reduced gravity environments of the Moon (1/6 g) or Mars (1/3 g), mass can appear deceptively light. This increases the susceptibility to inadvertent acceleration of objects. The use of helmets and hardened torso space-suits might reduce the likelihood and severity of serious thoracoabdominal injuries [20]. They would also induce chronic musculoskeletal strain, over-use injuries, and fail to protect against appendicular skeletal injuries however. Both NASA-Mir and EVA neutral buoyancy training experiences noted frequent small traumatic injuries and overuse syndromes especially of the upper extremities [11,23].

### The Physiology and Pathophysiology of Injury in Space

After a prolonged exposure to weightlessness, the injured astronaut will be at a physiologic disadvantage compared to patients on earth [6,21]. Changes likely to impair their ability to withstand injury [4] include: reductions in circulating blood volume, reduced red cell mass, cardiac atrophy, dysrythmias, reduced cardiac output, alterations in vascular tone and neuroendocrine function, loss of the protective bony mass, and possible immune suppression [3,4,7,24-28]. Fluid redistribution and diuresis result in a 10–23% volume reduction (equivalent to Class I ATLS hemorrhage on earth) even before the occurrence of an injury [6,22,24]. As a result, cardiac reserve is reduced and the autonomic nervous system is reset with greater beta receptor sensitivity. This could potentially prevent appropriate vasoconstriction[6,20,24]. Limited evidence also indicates that basic wound healing is impaired [29]. More specifically, histologic and tensiometric data from rat abdominal incisions performed during shuttle flights reveal greater inflammatory responses, increased fibroplasia, abnormal collagen deposition, and reduced stress loading capacity.

Loss of bony density during weightlessness appears proportional to the length of the flight and fails to display a self limiting plateau [30]. This may greatly increase the risk of fractures. In addition to appendicular fractures, degradation of the thoracic cage is concerning because it provides significant protection to vital viscera. On earth, fractures are the primary cause of more than half of all admissions for trauma [31]. While not typically life-threatening by themselves, serious long-bone fractures would be disastrous for a mission. Bone healing in space reveals impaired callus formation and reduced angiogenesis [32,33]. In true weightlessness bony integrity might not be absolutely required for ambulation however. Immobilization could also be obtained using simple air or thermoplastic casts/splints [22]. Whether the lack of gravitational loading would lead to mal- or disunion remains unknown. The risk of fat emboli with movement of unfixed bones is unclear as well. Without advanced techniques for bony fixation and gravitational loading for normal bone healing, even previously healthy astronauts may be permanently disabled.

The internal atmosphere of a space vehicle is likely to be populated by an increased number of virulent microbiological flora when compared to earth. Spaceflight investigations have reported that bacterial growth appears greater in weightlessness compared to terrestrial controls [34]. Thicker cell walls with higher minimal inhibitory requirements for antibiotics in common pathological bacteria have also been encountered [35,36]. Bacteria collected on the crew of the Apollo-Soyuz Project exhibited increased antibiotic resistances compared to pre and post-flight [37]. Whether spacecraft and their inhabitants can be adequately shielded from radiation and its effects on rapidly proliferating cells also remains unknown [38].

### Trauma Care in Space: A Unique Pre-Hospital Paradigm and a Test-Bed for Novel Life-Saving Technologies

In the United States, rural trauma mortality is up to 50% greater than in urban settings [39,40]. ECMs are expected to multiply this geographic differential several million times. With diminished physiologic health, more numerous and virulent microbiologic pathogens, potentially inexperienced care providers, and limited equipment, the outcome of serious trauma in space may seem to be a foregone conclusion. Fortunately the nature of these missions allows for medical preparation in a manner most prehospital care does not. Interplanetary space exploration represents the ultimate remote location, but one in which the most advanced medical technology of our society might be pre-positioned.

The distinct challenges inherent in providing medical care in space have not only led to unique solutions for space medicine, but also for terrestrial healthcare as well [41,42]. The biomedical support provided to the early manned exploration of space was a momentous achievement that has benefited countless patients requiring physiologic monitoring in terrestrial intensive care units [3]. The presence of an advanced ultrasound (US) machine onboard the ISS may provide another series of terrestrial spin-offs [43-45]. Currently the ISS has no radiography, computed tomography (CT), or magnetic resonance (MR) imaging, but does have US capability. This paradigm has driven a series of innovative investigations into the capabilities of sonographic techniques to aid the critically injured in space [43,45-48]. It is likely that future needs will also prompt the rapid development of other medical technologies.

### Space Physicians and Training Requirements for the Space Surgeon

The Crew Medical Officer (CMO) onboard the ISS is not currently required to be a physician, and is typically a specialist in a non-medical discipline with 60 hours of medical training [4]. While a broadly trained surgical specialist would be the obvious choice to deliver complex interventions in trying circumstances, there is no guarantee that a surgeon, or even a physician will be selected for future long duration missions. The CMO in such a scenario will be a mission specialist with other non-medical duties that might include psychological support and evaluation. This individual will also be vulnerable to disease and injury themselves, and will require an assistant to address more complex medical problems. The initial training and ongoing maintenance of competence of the medical/surgical officer is of paramount importance. Although still controversial, a broad range of competences incorporating many of today's specialties will be required. While the best pre-requisite training and planned curriculum of space medical/surgical training are unclear, they will largely be determined by the capabilities planned for a particular mission. If telemedical support is available, on-board technical skills will have greater value than knowledge, given the current ease of transmitting information [6]. Active research and development in telementoring and telerobotics is currently addressing this disparity [46,49]. In missions beyond LEO, tele-support will be extremely limited. As a result, acute medical care will need to be entirely autonomous.

### The Initial Resuscitation of Traumatic Injury in Space

The initial resuscitation of any critically injured patient is guided by ATLS, which addresses the most immediate threats to life first. This occurs whether the victim is in the simplest rural hospital or the largest tertiary care centre. It is generally perceived that applying basic ATLS principles in space is largely possible even with the limited resources currently aboard the ISS [50]. Securing an adequate airway is a critical first step in any resuscitation. It has been shown in parabolic flight that definitive control of the airway is possible via endotracheal intubation, insertion of a laryngeal mask, and even with a tracheostomy [50]. A focused sonographic examination of the chest after intubation offers a validated method of remotely and/or automatically confirming correct endotracheal tube positioning [51,52].

Pneumothoraces (PTXs) are the most common serious intra-thoracic injury following blunt trauma [53,54] and are a notable cause of preventable death for which relatively simple interventions may be life-saving [55-58]. PTXs are also a condition that may be exacerbated by the hypobaric stresses associated with EVA [7,57]. Tension pneumothoraces have been easily decompressed with both needle and tube thoracostomies in parabolic flight [4,50]. In many cases however, it may be more difficult to detect hemo-pneumothoraces than to actually treat them. This is certainly true aboard the ISS, where less experienced care providers will attempt auscultation of breath sounds in an environment with very loud ambient noise[18,44,59]. The problematic issue of PTXs demonstrates a case where an operational need stimulated a basic and applied research program that investigated the utility of US in detecting the presence of PTXs via abnormal pleural movements [44,60]. Because PTXs are a pleural based disease, US has proven remarkably sensitive, both in weightlessness and terrestrially. This fact is now receiving additional attention in terrestrial trauma care as well [59,61-63]. Ultrasound is a very user-dependant tool, and one in which CMOs are unlikely to be highly proficient. Recognizing this reality, Dulchavsky and colleagues have created a research program to enable minimally trained care providers to use US autonomously or with remote guidance [43,45,64].

Although increasingly controversial, fluid administration has long been considered an integral part of most initial resuscitations [21,65]. Based on parabolic flight experience, both large volume and titrated constant fluid infusions should be easily administered in weightlessness [4,47]. This requires degassed solutions administered with constant pressure infusions [4,47]. Severe injuries (e.g. burns) would quickly exhaust on-board crystalloid fluids however, necessitating the capability for the on-board generation of medically suitable fluids from processed water.

### Hemorrhage control in space

All of the previously mentioned basic resuscitative techniques are within the clinical capabilities of the CMO skill-set. Unfortunately, arresting hemorrhage is far more critical than administering fluids however. Excess fluid dilutes clotting proteins, exacerbates bleeding, and induces hypothermia. The easiest source of bleeding to address should be external or compressible hemorrhage. In operational settings, this is not always a simple endeavor. Even in recent military conflicts, many healthy soldiers have bled to death from wounds that should have been easily controlled. The simplest and safest maneuver is direct pressure [66]. Tourniquets are important in operational settings and can be used temporarily while other resources are activated [20,67,68]. More effective bandages and dressings for hemorrhage control have also been developed in the past decade [69-72]. Tissue sealant bandages, with ingredients very similar to fibrin glue, allow significantly less blood loss compared to both standard gauze [73].

### Truncal Hemorrhage

A current weakness in the commitment to life-saving trauma interventions for injured astronauts is addressing the potential for intracavitary hemorrhage control. This most commonly implies abdominal or thoracic surgery to control internal bleeding. Unfortunately, in 99% of civilian hemorrhagic deaths, external pressure is insufficient because the site of bleeding is truncal or non-compressible [74]. On earth, these incidents have been labeled as the leading cause of potentially preventable, injured-related death world wide [75]. They account for 80% of early hospital deaths, and are most frequently abdominal in origin [76]. Sauaia and colleagues examined all trauma deaths in Denver and found that while brain injuries were most frequent (42%), they were followed closely by hemorrhage (39%)[16]. A simple extrapolation suggests that if head injuries were not encountered in a space setting (i.e. hardened helmets are worn for all EVAs), cavitary hemorrhage would constitute 76% of all deaths.

Sites of massive internal hemorrhage may be intrapleural, intraperitoneal, and/or retroperitoneal. On earth, the use of US to quickly localize such bleeding has become standard practice [77-79]. Studies in both parabolic flight and true space suggest that both thoracic and abdominal blood will remain quickly detectable in weightlessness with accuracy. Controlled porcine studies in parabolic flight revealed that abdominal sonography appears at least as sensitive for the detection of intraperitoneal and intrathoracic fluid in weightlessness. This is presumably related to the enhanced importance of surface tension forces in determining intra-abdominal fluid behavior in the absence of gravity [47,80,81].

### Non-Operative Management

The management of many serious thoracoabdominal injuries has changed radically over the last several decades with a progression from routine exploration to careful observation [82]. Although accurate imaging on earth supports this approach in many cases, these injuries should always be managed in a setting with immediate access to critical care and operative abilities [82]. When non-operative management is unsuccessful it is usually because of early recurrent hemorrhage, or the late formation of bilomas, urinomas, asbcesses, or pseudoaneurysms. Many of these delayed complications can be successfully treated with percutaneous treatments. This procedure has been demonstrated to be feasible during parabolic flight [83]. Because of the adoption of these non-operative strategies, surgical intervention is typically triggered by ongoing hemorrhage manifested as physiologic failure or shock. As a result, these advances have relied on the ready availability of experienced surgeons with the capabilities to intervene if a patient's clinical condition worsens [6,84]. In austere environments such as space, it may be desirable to intervene in a controlled fashion prior to such gross physiological instability. Implicit in such a decision is the availability of safe and effective anesthetics. Inhalational anesthetics have theoretical limitations in space related both to weightless and the use of gasses in a small closed-loop environment [6]. Completely intravenous anesthetic regimes have been used in parabolic flight, but would require yet another CMO skill-set.

### Predicting the need for surgical intervention

Early bleeding or peritonitis may necessitate surgery given the lack of critical care in space. Satava has stressed the information systems integration benefits of total body imaging (holomers)[85]. While CT & MRI clearly yield superior images, any 3-dimensional (3D) imaging technique supported by decision support software might predict the outcome of an injury based on the real-time assessment bleeding. Hoyt estimated survival windows reliant on terrestrial physiology and hemorrhage volumes to be 2 hours based on 25 mls of bleeding per minute. If bleeding exceeded 100 mls/minute, death would ensure within 30 minutes [86]. 3D US is a technical development that displays an improved ability to quantify volumes compared to 2D [87,88]. It might also be combined with US contrast media to follow real-time hemorrhage [89]. Accounting for altered space physiology, on-board computers could notify the CMO when death would be inevitable without intervention. This would simplify an otherwise agonizing decision.

### Surgical interventions

The evidence suggests that standard surgical approaches would be possible in weightlessness as long as the key principles of restraint of the operators, subject, and equipment were followed [4,90,91]. A number of investigators have evaluated various surgical preparation and procedures in the weightlessness of parabolic flight by staging procedures to conform to the brief time windows. Procedures that have been evaluated include surgical site preparation, opening and closing wounds, laparotomies, repair of major abdominal vascular injuries, excision of the adnexae, observing the behavior of hepatic, splenic, renal and renal-vascular injuries [22,47,91-93], as well as the microvascular repair of rodent arteries and nerves [94].

A small number actual surgical procedures have been performed in the true space of orbital flight. Although dissections were performed aboard the STS-58 Spacelab Life Sciences mission in 1993, the most complex operations occurred during the 1998 Neurolab STS-90 Life Sciences mission. To gain an improved understanding of nervous system development, complex procedures included the administration of general anesthesia, hemostasis, control of surgical fluids, operator restraint, and manipulation of surgical instruments [95,96]. These investigations remain the pinnacle of actual surgical technique in true space. None of the subjects were human, nor required nursing care back to full mission critical status however. This would obviously be crucial for the success of any long-duration space mission. In an ECM, any requirement for convalescence and rehabilitation will also be a critical concern that should be considered in any treatment algorithm [6].

### Minimally invasive surgery

Minimally invasive surgical (MIS) techniques utilize the general principles of minimizing access incisions and completing the operative procedure within a patients' internal cavities [97]. MIS for trauma in space has been found to be feasible in weightlessness during parabolic flight[28,31]. Potential benefits include minimizing post-operative morbidity, shielding the cabin environment from biological components, protecting the patient from environmental particulates, maintaining thermal stability, and facilitating blood collection and autotransfusion. Contrary to previous predictions, the ability to perform laparoscopy in weightlessness did not appear to be any more difficult than in the 1-g environment [98,99]. Furthermore, the abdominal cavity appeared to change from a flattened oval to a rounded shape. This created more volume to operate. MIS has therefore been proposed as a potential option to reduce surgical morbidity in space [100,101]. Investigators have also considered potential techniques to reduce the skill-level required for the delivery of MIS in operational settings. Broderick and colleagues [100] evaluated simulated hand-assisted laparoscopy in parabolic flight. Dulchavsky has also recently evaluated the potential for telementored non-surgeons to perform simple laparoscopic drainage procedures in parabolic flight using mini-laparoscopes to further reduce surgical injury.

### Unanswered Questions Regarding the Safety of MIS in Prolonged Weightlessness

Despite the potential advantages to performing standard laparoscopy, significant safety and practicality concerns for these techniques in a closed space-craft environment remain. Even in tertiary care centers, MIS still remains limited in scope for the management of acute trauma [102,103]. It is also fundamentally unclear if an injured astronaut could tolerate the increase in intra-abdominal pressure caused by introducing carbon dioxide gas associated with MIS [104]. Many adverse physiologic consequences of raised intra-abdominal pressure have recently been appreciated in the critically ill [105-107]. As a result, physicians routinely attempt to avoid this condition in terrestrial practice by leaving the abdominal compartment open [108,109]. Considering that even healthy astronauts will have diminished blood volumes and cardiac deconditioning [6], there are serious concerns that injured or septic hypovolemic astronauts could tolerate the physiologic stresses of laparoscopy [104].

### Gasless Laparoscopy

Gasless laparoscopy or the performance of intra-abdominal MIS without positive pressure insufflation is of particular interest for space scientists. On earth, "abdominal wall lift devices" have been used to eliminate the need for gas insufflation [110]. These devices have not gained practical acceptance due to limitations on the resultant domain. Limited experiences in parabolic flight suggested that spontaneous abdominal wall changes occurred in weightlessness, with an inherent increase in the available domain even without insufflation [99]. Unfortunately, this change in abdominal wall shape did not equate to a functional window during laparoscopic surgery in parabolic flight [111]. This was primarily because the intraperitoneal viscera "floated" within the abdomen and therefore reduced the operating domain.

### The Damage Control Philosophy

Although MIS has many theoretical benefits, early open exploratory surgery in space may be necessitated by catastrophic conditions such as shock or septic syndrome prior to a specific diagnosis. As early as 1983, a council of trauma surgeons, space physicians, and biomedical engineers identified the ability to perform laparotomy as the minimum desirable surgical capability to save lives before transfer to earth [112]. Damage control (DC) surgery refers to a philosophy of completing only the most necessary components of a procedure via the simplest methods. This technique is used to address problems beyond either the patient's physiologic reserve, or the "local capabilities" of a care setting [113,114]. Fortunately, DC techniques are technically "easier" than most formal surgery [113,115]. Placing "packs" around bleeding solid organs and leaving the abdomen "open" are the most basic elements of DC. While psychologically daunting, incising the anterior abdominal wall to access the peritoneal cavity is technically simple. It may also convert non-compressible bleeding into directly compressible visceral hemorrhage. Non-physicians have reportedly performed this successfully [116]. Fibrin glue, or tissue sealant has been also been formulated as a foam which can be easily administered by non-experts. Foam and sealant dressings have been found to be more effective than standard surgical packing in intracavitary bleeding [117].

The DC philosophy also extends to orthopedic injuries. These techniques emphasize the early fixation of long-bones using external fixators (vs. internal). They are simple, and rapid with minimal physiologic stress, and reduced anesthesia requirements [118-120]. While they are considered an interim step towards complicated internal fixation on earth, they might allow mobilization in a zero or reduced gravity environment. Diagnosis and real-time anatomic reduction of long bone fractures has been demonstrated with US [121-123]. Film-less radiography is another option that uses an inflatable arm for beam orientation [124].

### Reality check: Nursing and Recovery in Space

The DC approach to severe trauma has tremendous appeal and potential utility for evacuating a seriously injured astronaut from LEO. Although it might also be applicable to a lunar mission, DC would be impractical during a mission to Mars where return to earth is impossible. In such a setting, managing an open abdomen in space would create potentially insurmountable medical, logistical, and psychological problems. On earth, the prolonged use of intraperitoneal packs is itself associated with greater morbidity and mortality when the duration exceeds 72 hours [125,126]. This is likely to be a greater problem in space with increased microbial virulence and human immune suppression.

### Vascular Access and Intra-luminal Interventions

Accessing vascular pathology via an intra-luminal route is revolutionizing care in vascular surgery, cardiology, and trauma. Access to the central circulation would also allow optimized hemodynamic measurements, hemodynamic support, interventional angiographic guided therapies, and provide the most efficient location to administer pharmacologic therapy. Because of the numerous potential complications related to central vascular access (including misinterpretation of measurements), this intervention should ideally be automated. Although Doppler guided needles have been available for a decade [127], fully automated vascular target identification using smart ultrasound-guided "bibs" are in development [128]. In the future, full robotic control may be possible.

Early measurement and correction of reduced central venous pressures has been recommended before administering anesthetics in space, as well as in the early intervention of septic shock on earth [24,129]. With central venous access, vasopressor and inotropic medications that may be required in the treatment of space-altered cardiovascular physiology may be given with an improved safety profile. Heparin bonded extracorporeal circuits placed after multi-system trauma appear safe for both rapid rewarming [120], and providing hemodynamic support [131] after severe trauma. Hemorrhage control via the arterial system in the form of interventional radiology is now a standard approach to surgically inaccessible bleeding. As a result, it is now being used considerably earlier for injured patients [132,133]. An autonomous and image guided robotic capability would be highly desirable.

### Pharmacological adjuncts

Even with generous administration of blood products, coagulopathy is often a determining factor in survival after injury [134]. If blood products are unavailable in space, pharmacologic adjuncts (i.e. "trauma cocktail") may play a significant role. Potential adjuncts include vasopressin, as well as anti-fibrinolytics such as aprotinin, transexamic acid and recombinant Factor VIIa [20,21,135].

### Surgical robotics and resuscitative surgery in space

Although not broadly accepted, the use of surgical robots has become common in certain subspecialties. Current robots often add time to procedures and require additional power while increasing fidelity, precision, and the potential for tele-operation [136]. Ongoing investigations have shown the feasibility of operating across continents and to undersea space analogue environments [49,137]. While these techniques might be valuable for LEO, they would be impractical for ECMs due to the great distances between earth and even our closet planetary neighbor (Mars). This would entail round-trip electronic delays from 8 to 40 minutes [6,138]. As a result, any robot in space would require guidance either by an experienced surgeon, or to be autonomous with image-guidance. It is uncertain which technology will ultimately provide the optimal balance of image-fidelity, reliability, minimal-mass, and ease of operation, but CT scan, MRI, and ultrasound are all potential modes. Functional MRI has already been guided in real-time with concurrent parameter control and automated slice positioning/tracking [139].

### Suspended Animation: The Ultimate Paradigm Shift for Trauma Care

The logistical realities on an ECM remain extensive and possibly unrealistic with regards to surviving complex injuries. In such a reality, euthanasia will need to be discussed and understood. As emphasized, all currently accepted strategies for preventing death after major trauma focus on quickly restoring blood flow to minimize both tissue ischemia and reperfusion injury. Recent developments in our understanding of the basic science of suspended animation (SA) hint at a completely different approach. SA has been defined as the therapeutic induction of a state of tolerance to temporary complete systemic ischemia [140]. This results in a dramatic reduction in both energy production (metabolism) and energy consumption (cellular activity) [141]. More broadly, this concept involves removing oxygen from an injured person to preserve cellular integrity and allowing either delayed repair or simply preservation [141]. Significant research has been invested to identify potential SA techniques. Profound hypothermia using extracorporeal circulation has been successful in keeping severely injured dogs pulseless for 2 hours before returning them to normal function via resuscitation [142-144]. The obvious limitation is the need for a cardiac bypass pump to rapidly induce ultra-profound hypothermia (5°C). If major vascular access were available during an ECM this might offer the opportunity to quickly cool a patient, providing a window for multiple virtual reality rehearsals prior to an on-board repair in a bloodless field.

Hydrogen sulfide is a specific, reversible inhibitor of oxidative phosphorylation, that profoundly inhibits the metabolic rate and depresses the core body temperature of mammals [145]. It reduces the metabolism of mice by 90% after six hours without inducing obvious behavioral or functional problems when reversed [145]. If this approach could be safely extended for sufficiently long periods in human astronauts, on-board medical care would be greatly simplified. As a result, all major pathology would be "stored and forwarded" to the eventual return to earth. Multiple casualties or illnesses impacting the ability of the crew to manage the mission are an obvious limitation to this strategy.

## Conclusion

Given the risks and isolation inherent in long duration spaceflight, a clever surgeon and/or surgical capability will be required onboard a Mars exploration vessel. This is essential because telemedicine is fundamentally limited by the speed of light. Although not yet technologically available, the operator does not have to be human if an image guided robot were present. In the mean time, a broadly-trained surgically capable emergency/critical care specialist with innate capabilities to problem-solve and improvise would be desirable as a CMO. An ECM will present a truly unique paradigm, unlike any other medical setting on earth. It will be the ultimate remote setting, and hopefully one in which the most advanced of our societies' technologies can be pre-positioned to safeguard precious astronaut lives. Like so many previous space-related technologies, these developments will also greatly improve terrestrial care on earth.

## Competing interests

The authors declare that they have no competing interests.

## Authors' contributions

Each of the authors (AWK, CGB, MC, DRW, SEP, KLM) was critically responsible for development/conception of content, analysis of data/literature, as well as manuscript writing/revisions.

## Note

The topics described in this manuscript are derived from a consensus conference outlining treatment capabilities for injured astronauts in extended duration spaceflight.
